# Editorial: Brain Oscillations in Human Communication

**DOI:** 10.3389/fnhum.2018.00039

**Published:** 2018-02-07

**Authors:** Johanna M. Rimmele, Joachim Gross, Sophie Molholm, Anne Keitel

**Affiliations:** ^1^Department of Neuroscience, Max Planck Institute for Empirical Aesthetics (MPG), Frankfurt am Main, Germany; ^2^Institut für Biomagnetismus und Biosignalanalyse, Universitätsklinikum Münster, Münster, Germany; ^3^Departments of Pediatrics and Neuroscience, Albert Einstein College of Medicine, Bronx, NY, United States; ^4^Centre for Cognitive Neuroimaging, University of Glasgow, Glasgow, United Kingdom

**Keywords:** speech perception and production, non-verbal Communication, brain rhythms, neurobiology of language, MEG source analysis, communication disorders

This Research Topic featured 15 articles from a wide range of research areas related to human communication. All contributions focus on rhythmic brain activity as opposed to, for example, event related potentials or functional imaging approaches. Rhythmic brain activity has been shown to be of immense importance for the temporal coordination of neural activity and, consequently, for all aspects of cognition and behaviour (Buzsaki and Draguhn, 2004; Wang, 2010). In this editorial, we summarise the research on rhythmic brain activity in language and communication that appeared in this Research Topic (see Table 1).

**Table 1:** Papers in this issue categorized along methodological and conceptual dimensions.

**Authors**	**Type**	**Group**	**Method**	**Modality**	**Domain**
Aubanel et al.	Original research	Healthy human	Behavioural	Auditory	Speech perception
Benítez-Burraco and Murphy	Hypothesis and Theory	ASD	All	Auditory Visual	Language perception
Biau and Soto-Faraco	Perspective				
Jenson et al.	Original research	Healthy human	EEG	Auditory	Speech perception, production
Jochaut et al.	Original research	ASD	EEG, fMRI	Auditory	Speech perception
Lewis et al.	Perspective	Healthy human	All, MEG	Auditory Motor	Speech processing
Murphy and Benítez-Burraco	Hypothesis and Theory	Schizophrenia	All	Auditory Visual	Language perception
Nourski et al.	Original research	Epilepsy	ECoG, electrode arrays	Auditory Visual Motor	Speech perception, production
O'Connell et al.	Original research	Macaque	Electrode array	Auditory	Speech perception
Piai et al.	Original research	Healthy human	MEG	Auditory	Speech production
Ruhnau et al.	Original research	Healthy human	tACS	Visual	Flicker
Symons et al.	Review	Healthy human	EEG, MEG	Emotion Auditory Visual	Emotion Non verbal communication
Tognoli and Kelso	Hypothesis and Theory	Healthy human	EEG, MEG	Auditory Motor	Social cognition, communication
Zhou et al.	Hypothesis and Theory	Healthy human	EEG, MEG, LFP	Auditory	Speech, music perception
Zoefel and VanRullen	Review	Healthy human	All	Auditory	Speech perception

*ASD, autism spectrum disorder; EEG, electroencephalography; ECoG, electrocorticography; fMRI, functional magnetic resonance imaging; LFP, local field potential; MEG, magnetoencephalography; tACS, transcranial alternating current stimulation*.

## Perception of spoken language

Low-frequency (delta, theta) neuronal oscillations (and the coupling with gamma-band oscillations) have been shown to have a crucial role in speech perception, particularly in the segmentation of the continuous acoustic stream into linguistically meaningful units (Giraud and Poeppel, 2012; Ding and Simon, 2014; Ding et al., 2016; Keitel et al., in review). By now, many studies have shown that low-frequency neuronal activity in auditory cortex tracks the slow energy fluctuations in the speech acoustics (Luo and Poeppel, 2007; Gross et al., 2013; Rimmele et al., 2015; Keitel et al., 2017a). The functional relevance of this neuronal phase alignment, however, and whether it actually reflects the entrainment of endogenous neuronal oscillations to a speech signal (as opposed to event related potentials) are controversial.

O'Connell et al. shed light on the tonotopy of multiscale neuronal entrainment in auditory cortex (A1) by using single cell recordings in macaques. They provide evidence for multiscale entrainment to clicks presented at regular intervals in the gamma and delta range. Particularly, neurons in the region of 11–16 kHz on the tonotopic maps of A1 aligned their excitability to the attended sounds, while the remaining part of A1 showed response suppression. The findings suggest a function of cortical entrainment to a rhythmic stimulation in the selection and processing of attended sounds (O'Connell et al., 2014), which might be at play in speech perception.

A crucial question is which aspects of the speech signal trigger the low-frequency phase alignment to the speech acoustics that are involved in speech comprehension. Aubanel et al. investigated this by isochronously re-timing temporally distorted speech with anchor points either at syllable onsets (linguistic cues) or at amplitude envelope peaks (acoustic cues). They showed that speech comprehension benefits most from linguistically motivated cues, that is, a re-timing at stressed syllable onsets.

The extent to which low-frequency neuronal phase alignment can be modulated by higher-level processes is controversial (Haegens and Golumbic, 2017; Teng et al., 2017). By reviewing previous literature, Zoefel and VanRullen discuss the contribution of high-level processes to the cortical low-frequency entrainment to speech. Particularly, based on high-level modulations of speech processing, as well as the finding of entrainment in the absence of low-level rhythmical cues (Zoefel and VanRullen, 2015, 2016) they suggest that the theta phase alignment indicates oscillatory entrainment and not mere stimulus-driven responses caused by a rhythmic stimulation (cf. Keitel et al., 2014). The authors discuss the functional role of cortical entrainment with respect to speech intelligibility and provide theoretical considerations to integrate stimulus driven effects and top-down modulations of speech processing into a unified model.

Lewis et al. focus on a specific aspect of high-level processes in spoken language comprehension. They review current research on the role of beta-band oscillations in sentence processing. This research suggests a function either in sentence-level top-down predictions about the up-coming linguistic input, or in indicating the maintenance of the current processing mode, relevant for deriving the meaning of a sentence (Lewis and Bastiaansen, 2015; Lewis et al., 2015). In this review, they additionally present preliminary magnetoencephalography (MEG) data, supporting the latter interpretation.

## Speech production and neuronal oscillations

Although cortical oscillations have been shown to be involved in sensorimotor coordination (Arnal and Giraud, 2012), which is vital for speech production, the exact function of oscillations in speech production is little understood. Jenson et al. investigated the temporal dynamics of alpha-band activity in the auditory posterior dorsal stream during speech production and perception, using a novel analysis method (combining independent component analysis and event related spectral perturbations) in electroencephalography (EEG) recordings. Together with previous findings by Jenson et al. (2014), these results show the temporal dynamics of the anterior and posterior dorsal stream during speech perception and production. In sum, their findings suggest a crucial role of alpha oscillations in sensorimotor interactions that allow monitoring the speech production through efference copies from the motor system.

The ability to control our speech output and withhold planned speech is critical during communication, as we need to time the turn-taking of the interacting partners (Wilson and Wilson, 2005). In an MEG study, Piai et al. investigated neuronal activity during the withholding of planned speech. They provide evidence for a two-fold mechanism. First, alpha-band desynchronisation in occipital brain areas might indicate the task-specific allocation of attention during the withholding of speech. Second, increased frontal beta-band activity during the withholding of speech most likely indicates the maintenance of the current motor or cognitive state, i.e., maintaining the planned verbal response (Engel and Fries, 2010; Piai et al., 2015; Rimmele et al., in review).

## Social cognition and communication

In a more natural communication setting, Nourski et al. tested speech production and perception during a conversation between epilepsy patients and an instructor, using electrocorticography (EcoG) recordings. They found no difference in high gamma activity in the auditory core cortex when listening to self-produced speech vs. the speech of others. However, gamma-activity was reduced in non-core areas when participants listened to their own speech. The findings indicate that signals from self-produced speech are differentiated from speech of others at higher non-core auditory processing areas, and high gamma oscillations play a role in these processes (Nourski, 2017).

Tognoli and Kelso approach social cognition and communication beyond the individual brain, by pursuing the hypothesis that social interaction results in the phase-locking and coupling of neuronal activity across brains (e.g., Dumas et al., 2010). They theoretically underpin a neuromarker approach to social cognition, review findings from dual-EEG recordings (Tognoli et al., 2007a,b), and discuss them in the context of previous research on social cognition. In sum, neuromarkers in the alpha, mu, kappa and phi bands seem to be differentially involved in simultaneous action and perception processes (e.g. tango dancing) and the alternating perception and production of social behaviour (e.g., imitating someone).

## Oscillations in non-verbal communication

For successful interpersonal communication, it is crucial to detect and identify emotional expressions from auditory, visual, and audiovisual information (Jessen and Kotz, 2011; Kotz et al., 2013). Here, Symons et al. review the available literature focussing on oscillatory mechanisms. They conclude that theta- and gamma-band synchronisation most consistently reflect the processing of emotional expressions across sensory modalities (e.g., Knyazev, 2007; Luo et al., 2008). On the other hand, oscillations in the delta-, alpha-, and beta-bands have also been implied in the processing of other's emotions, but their role is less consistent across modalities and tasks.

Another important non-verbal aspect of speech is the use of gestures (Hubbard et al., 2009; Biau and Soto-Faraco, 2013). Based on the previous finding that hand gestures phase-reset ongoing neural oscillations (Biau et al., 2015), Biau and Soto-Faraco discuss the role of beat gestures in audiovisual speech processing. They conclude that beat gestures promote a cross-modal phase reset at important word onsets, which might facilitate the segmentation of the speech stream.

## Pathological changes to communication in autism and schizophrenia

Many psychological and neurological conditions also affect the production or perception of language (e.g., Uhlhaas and Singer, 2006). Jochaut et al. investigated the response to continuous speech in individuals with and without autism spectrum disorder (ASD), using concurrent EEG and fMRI. They report anomalies of theta and gamma oscillations in the left auditory cortex in ASD participants, as well as altered functional connectivity between auditory and other language cortices. Furthermore, the theta/gamma coupling predicted verbal impairment as well as ASD symptoms.

Benítez-Burraco and Murphy take a different approach to language deficits in ASD. In their theoretical article, they propose to relate genetics to the pathophysiology of ASD by studying oscillatory mechanisms for language processing in the autistic brain. They note that candidate genes for ASD are overrepresented among the genes that played a role in the evolution of language and brain oscillations, thereby bringing together these different methodological approaches.

In a second theoretical article, Murphy and Benítez-Burraco (2016) take a similar approach toward understanding the relationship between genes and language deficits in schizophrenia. Here, they suggest that the language deficits in schizophrenia seem to be rooted in the evolutionary processes that brought about modern language. This evolutionary account and the common oscillatory profiles of language deficits in schizophrenia and ASD are further described in a recent article (Murphy and Benítez-Burraco, 2016).

## Methodological considerations and innovations

This Research Topic also featured predominantly methodological articles that could advance research into communication processes. Ruhnau et al. used a novel combination of concurrent transcranial alternating current stimulation (tACS) and frequency tagging. They carefully tease apart source-level tACS effects and steady-state responses (SSRs) in the MEG, by using a new method to reconstruct sources of SSRs that are unaffected by the strong tACS artifact (Neuling et al., 2015). Frequency tagging is a potentially fruitful approach for studying speech processing (Buiatti et al., 2009), and this proof-of-principle study opens up new possibilities to combine frequency tagging, tACS, and MEG.

Finally, Zhou et al. discuss the implications and limitations of the often used Fourier analysis to study low-frequency neural entrainment. They conclude that true low-frequency entrainment results in a peak in the power spectrum at the fundamental frequency (the lowest frequency produced by an oscillation), and describe how the phenomenon of higher harmonics can be interpreted.

In conclusion, the studies included here review, highlight, and specify the role of distinct cortical oscillations in practically all processes related to human communication.

## Author contributions

All authors listed have made a substantial, direct and intellectual contribution to the work, and approved it for publication.

### Conflict of interest statement

The authors declare that the research was conducted in the absence of any commercial or financial relationships that could be construed as a potential conflict of interest.
